# Regulation of cleavage embryo genes upon DRP1 inhibition in mouse embryonic stem cells

**DOI:** 10.3389/fcell.2023.1191797

**Published:** 2023-05-15

**Authors:** Shi-Meng Guo, Yi-Ran Zhang, Bing-Xin Ma, Li-Quan Zhou, Ying Yin

**Affiliations:** ^1^ Institute of Reproductive Health, Tongji Medical College, Huazhong University of Science and Technology, Wuhan, China; ^2^ Reproductive Medicine Center, Tongji Hospital, Tongji Medical College, Huazhong University of Science and Technology, Wuhan, China; ^3^ School of Basic Medicine, Tongji Medical College, Huazhong University of Science and Technology, Wuhan, China; ^4^ Center for Genomics and Proteomics Research, School of Basic Medicine, Tongji Medical College, Huazhong University of Science and Technology, Wuhan, China; ^5^ Hubei Key Laboratory of Drug Target Research and Pharmacodynamic Evaluation, Huazhong University of Science and Technology, Wuhan, China

**Keywords:** embryonic stem cell, DRP1, zygotic genome activation, totipotency, embryo

## Abstract

Dynamic-related protein 1 (DRP1) is a key protein of mitochondrial fission. In this study, we found that inhibition of activity of DRP1 led to increased levels of cleavage embryo genes in mouse embryonic stem cells (mESCs), which might reflect a transient totipotency status derived from pluripotency. This result indicates that DRP1 inhibition in mESCs leads to a tendency to obtain a new expression profile similar to that of the 2C-like state. Meanwhile, we also noticed that the glycolysis/gluconeogenesis pathway and its related enzymes were significantly downregulated, and the key glycolytic enzymes were also downregulated in various 2C-like cells. Moreover, when DRP1 activity was inhibited from the late zygote when cleavage embryo genes started to express, development of early embryos was inhibited, and these cleavage embryo genes failed to be efficiently silenced at the late 2-cell (2C) stage. Taken together, our result shows that DRP1 plays an important role in silencing cleavage embryo genes for totipotency-to-pluripotency transition.

## Introduction

Mitochondria are important and dynamic double membrane-bound organelles, and the balance of fission and fusion maintains their physiological functions (Youle and van der Bliek, 2012; Wai and Langer, 2016; Giacomello et al., 2020; Li et al., 2021). The guanosine triphosphatases (GTPases), which are well conserved in mammals, are responsible for this dynamic process (Youle and van der Bliek, 2012; Wai and Langer, 2016). As for the fission process, the dynamic-related protein 1 (DRP1), a member of the cytosolic dynamin family, is recruited by *Mid49*, *Mid51*, and *Mff* to function in mammalian mitochondria (Schmitt et al., 2018; Kleele et al., 2021; Li et al., 2021). Mitochondrial fission is essential for development, and it is demonstrated that the knockout of *Drp*1 in mice caused death of early embryos. In addition, the loss of *Drp1* seriously affects the maturation of oocytes and the establishment of maternal epigenome in preimplantation embryos (Udagawa et al., 2014; Li et al., 2021; Adhikari et al., 2022). Interestingly, the CRISPR-Cas9 system has been used to generate DRP1-knockout embryonic stem cells (ESCs), which showed significantly impacted mitochondrial metabolism and global gene expression profiles (Seo et al., 2020).

Mitochondria produce ATP for energy through oxidative phosphorylation (OXPHOS), which, in stem cells, is thought to rely mainly on glycolysis. Because of mitochondrial elongation, deletion of mitochondrial fission factors such as *Drp1*, *Mff*, or *Fis* resulted in increased OXPHOS and intracellular ATP concentration and decreased glycolysis (Seo et al., 2020). Recent studies have shown that mitochondria play an important role in the regulation of stem cell activity in terms of metabolism (Matilainen et al., 2017; Zhang et al., 2018; Ludikhuize et al., 2020). The intermediates in the mitochondrial metabolic pathway are closely related to key enzymes, which regulate chromatin and protein modification such as histones, methylation, and acetylation (Gafni et al., 2013; Carbognin et al., 2016; Matilainen et al., 2017; Zhang et al., 2018). Upregulation of mitochondrial transcripts and metabolism with activated STAT3 promotes the proliferation of mouse ESCs and the reprogramming of EpiSCs (Li et al., 2021). The inhibition of histone deacetylase (HDAC), which is dependent on NAD^+^, drove ESCs to a more naive state *in vitro* (Giacomello et al., 2020). In addition, sirtuin enzymes and mitochondrial metabolite NAD^+^ together regulate the post-translational modifications (PTMs) of proteins to orchestrate cell fate (Wai and Langer, 2016; Chini et al., 2021).

During early embryonic development, the developmental program depends on the degradation of maternal mRNAs and zygotic genome activation (ZGA) (Aoki et al., 1997; Srinivasan et al., 2020; Cheng et al., 2022). A great mass of transcripts and widespread changes in chromatin that occurred during the ZGA event complicated the regulatory module at this specific point. ZGA starts at the zygote stage and becomes robust at the 2-cell (2C) stage in mice, and thousands of 2C-specific genes (2C genes, ZGA genes, or cleavage embryo genes) (we refer to 2C gene in the following) are abruptly upregulated, such as *Zscan4* and *Dux* (De Iaco et al., 2017; Srinivasan et al., 2020; Grow et al., 2021). ZGA is controlled by different mechanisms including the expression of specific transcription factors (TFs) and the activity of RNA polymerase (Grow et al., 2021; Wang et al., 2022).

Our previous studies have shown that DRP1 deactivation led to mitochondrial abnormalities and thus affected the normal development of embryos (Li et al., 2021), but its effect on cell fate control remains unclear. In particular, mitochondrial division inhibitor-1 (Mdivi-1), which can inhibit the activity of DRP1, has been proposed to selectively inhibit mitochondrial division in mammals (Cassidy-Stone et al., 2008; Bordt et al., 2017). Herein, we use both mouse early embryos and mouse ESCs to investigate how DRP1 activity is involved in totipotency-to-pluripotency transition.

## Methods and materials

### Cell culture and treatment

The mouse ESC line AB2.2 was seeded on mitotically inactivated MEF feeder cells in the N2B27 medium with 2i, 0.4 μM PD0325901 (Stemgent, United States), 3 μM CHIR99021 (Stemgent, United States), and LIF (1000 U/mL) in tissue culture dishes (Buecker et al., 2014). Mdivi-1 was dissolved by DMSO for dilution to the designated concentration. ESCs were treated with 50 μM Mdivi-1 (MedChemExpress, United States) and cultured for a certain amount of time for further examination. The medium was changed every day, and the cells were cultured in humidified conditions with 5% CO_2_ at 37°C.

### Embryo collection and Mdivi-1 treatment

Female ICR mice (7–8 weeks) were injected with 10 IU of pregnant mare serum gonadotropin (PMSG, Sansheng, China). After 48 h, female mice were injected with 10 IU of human chorionic gonadotropin (hCG, Sansheng, China) and then let to mate with adult male ICR mice. Zygotes were collected from the oviduct ampulla of female mice with vaginal plugs 16 h later. Zygotes were cultured in the KSOM medium with or without 200 μM Mdivi-1 at 37°C in a humidified atmosphere of 5% CO2.

### Real-time PCR

Total RNA was extracted from 50 oocytes or 5 × 10^5^ cells using the TRIzol reagent (Invitrogen, United States), following the manufacturer’s instructions, and cDNA synthesis was completed using the Hifair 1st Strand cDNA Synthesis Kit (Yeasen Biotechnology Co., Ltd., China). RT-PCR was performed with the SYBR Green Master Mix (Yeasen Biotechnology Co., Ltd., China) with the ABI 7500 Real-Time PCR system (Applied Biosystems, United States). Mouse *Zscan4* (Forward: 5′- GAG​ATT​CAT​GGA​GAG​TCT​GAC​TGA​TGA​GTG-3′ and Reverse: 5′- GCT​GTT​GTT​TCA​AAA​GCT​TGA​TGA​CTT​C-3′), *Zfp352* (Forward: 5′- ACC​ACC​TCA​AAG​AAC​ACC​AG-3′ and Reverse: 5′- ACA​AGG​GAC​AAG​CGT​AGA​AC-3′), were normalized against *Gapdh* (Forward: 5′-TCT​TCC​AGG​AGC​GAG​ACC​C-3′ and Reverse: 5′-CGG​AGA​TGA​TGA​CCC​TTT​T-3′), and the quantification of the fold change was determined by the comparative CT method.

### Western blot

The samples (10^6^ cells/line) were lysed with the 100 μL RIPA buffer (Sangon Biotech, China), and the lysates were separated by SDS–polyacrylamide gel electrophoresis at 110 V for 60 min. Separated proteins were then transferred onto the nitrocellulose filter membrane at 350 mA for 60 min in ice. The membranes were blocked in PBS (Solarbio, China) with 1% (wt/vol) BSA (Sangon Biotech, China) for 1 h at room temperature and then incubated with primary antibodies overnight at 4°C. The primary antibodies against DRP1 (1:1000, 8570, CST, United States) and GAPDH (1:1000, 10494-1-AP, Proteintech Group, Inc., United States) were used. After washing three times, the membrane was incubated with HRP-conjugated goat anti-rabbit IgG (H + L) (1:5000, SA00001-2, Proteintech Group, Inc., United States). Solutions A and B of the ECL chemiluminescent solution (Affinity Biosciences, K002, United States) were mixed in a ratio of 1:1 to cover the membrane for incubation. The membrane was protected from exposure to light. Images were collected using a gel imager (Image Lab Software, Bio-Rad) in the dark.

### RNA-seq

For mouse ESCs, total RNA was extracted by the TRIzol reagent (Invitrogen, United States) from 5 × 10^5^ cells for each group, followed by standard RNA-seq library preparation (TruSeq RNA Sample Preparation Kit, Illumina). For late 2-cell embryos, 8–10 embryos were collected after 16 h *in vitro* culture for each group in lysis components with ribonuclease inhibitors, and amplification was further carried out by the Smart-Seq2 method by BGI Genomics Co., Ltd., China. Qualified libraries were loaded onto the Illumina HiSeq platform for PE150 sequencing. Raw reads were processed with cutadapt v1.16 to remove adapters and perform quality trimming with default parameters except for a quality cutoff of 20 and a minimum length of 20. Trimmed reads were mapped to the mouse genome (GENCODE release M23) using STAR with default settings. Reads were counted in exons of the mouse genome, using the STAR-quantMode GeneCounts setting. RSEM was used to calculate the FPKM value. Differentially regulated genes in DESeq2 analysis were defined as those which were more than two-fold increased or decreased with adjusted *p* < 0.05. Gene Ontology (GO) and Kyoto Encyclopedia of Genes and Genomes (KEGG) analyses were performed by Metascape (https://metascape.org). Volcano plot, heatmap, bar chart, and bubble chart were generated by R software.

### ChIP-seq

ChIP-seq was performed using a Hyperactive *In-Situ* ChIP Library Prep Kit for Illumina (pG-Tn5) (Vazyme, Nanjing, China), according to the manufacturer’s procedure with the appropriate antibodies H3K4me3 (9297, CST, United States), H3K27me3 (ab6002, Abcam, United Kingdom), and Pol II (39097, Active motif, United States).

### Statistical analysis

Data were presented as the mean ± SEM. All experiments were replicated more than three times. Statistical comparisons were made with the Mann–Whitney U test for the analysis of two groups. Analyses were conducted by SPSS 20 software (IBM). A *p*-value <0.05 (*p < 0.05) was considered statistically significant.

## Results

### Expression of cleavage embryo genes was increased in *Drp1*-KO ESCs

**FIGURE 1:**
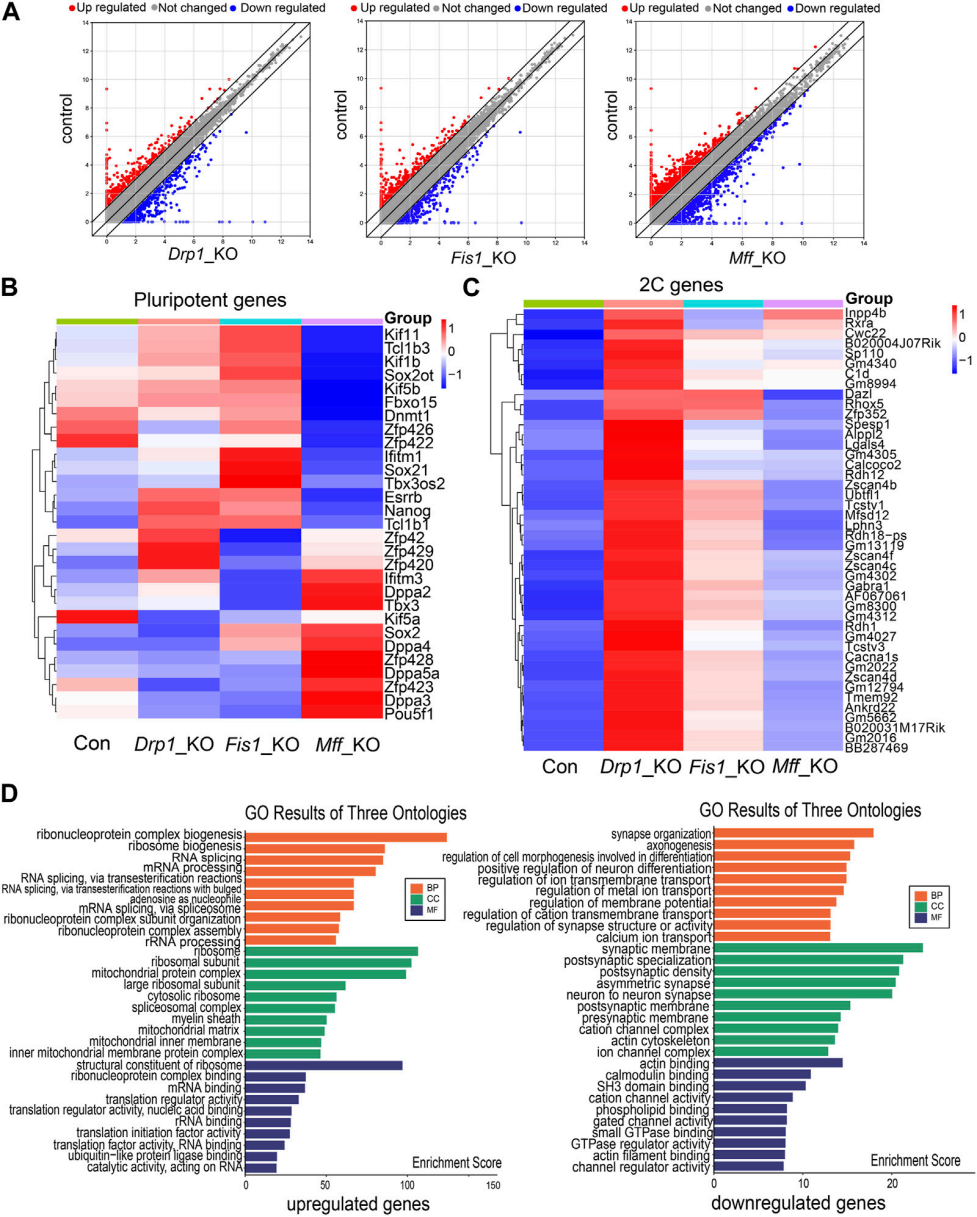
RNA-seq data analysis on ESC lines with knockout of various mitochondrial fission factors. **(A)** Scatter plots of differentially expressed genes in *Drp1-*KO ESCs (left), *Fis*-KO ESCs (middle), and *Mff*-KO ESCs (right) compared with control ESCs. Heatmap of differential expression of pluripotent genes **(B)** and 2C genes **(C)** in *Drp1-*KO ESCs, *Fis*-KO ESCs, and *Mff*-KO ESCs compared to the control group. **(D)** Gene Ontology (GO) enrichment analysis of upregulated (left) and downregulated genes (right) in *Drp1*-KO ESCs compared to control ESCs.

We first analyzed the RNA-seq data (GSE148959) on mouse ESCs with knocked-out mitochondrial fission factors (Seo et al., 2020). The results showed that deletion of mitochondrial fission factors, such as *Drp1*, *Mff*, or *Fis*, resulted in the change in gene expression in ESCs compared with the control group (Figure 1A). There were more than 700 differentially expressed genes in *Drp1*-KO ESCs, and nearly half of them were upregulated or downregulated (Figure 1A). There were about 1000 differentially expressed genes in *Mff*-KO ESCs and 600 differentially expressed genes in *Fis*-KO ESCs (Figure 1A). There was no significant difference in the expression of pluripotency factors such as *Oct4*, *Sox2*, and *Nanog* in *Drp1*-KO, *Mff*-KO, and *Fis*-KO ESCs, respectively, compared with the control group (Figure 1B). It was also reported that the pluripotency of the three knockout ESC lines was not affected (Seo et al., 2020). Next, we scrutinized the differentially expressed genes of the three knockout ESC lines (*Drp1*-KO, *Mff*-KO, and *Fis*-KO) and found that deletion of mitochondrial fission factors led to the upregulation of many 2C genes (cleavage embryo genes) (Figure 1C). In the *Drp1*-KO ESC line, 2C genes were mostly obviously upregulated (Figure 1C). GO analysis showed that DEGs were enriched in mRNA processing and mitochondrial metabolism in *Drp1*-KO cell lines (Figure 1D). The mitochondrial metabolic pathway is closely related to the pluripotent state of ESCs (Mandal et al., 2011; Rodrigues et al., 2015) and also participates in the regulation of 2C gene expression in ESCs and embryos (Nagaraj et al., 2017; Hu et al., 2020). These results suggest that the mitochondrial fission factor *Drp1* is involved in the regulation of 2C gene levels in mouse ESCs.

### Inhibition of DRP1 promoted expression of 2C genes in mouse ESCs

**FIGURE 2:**
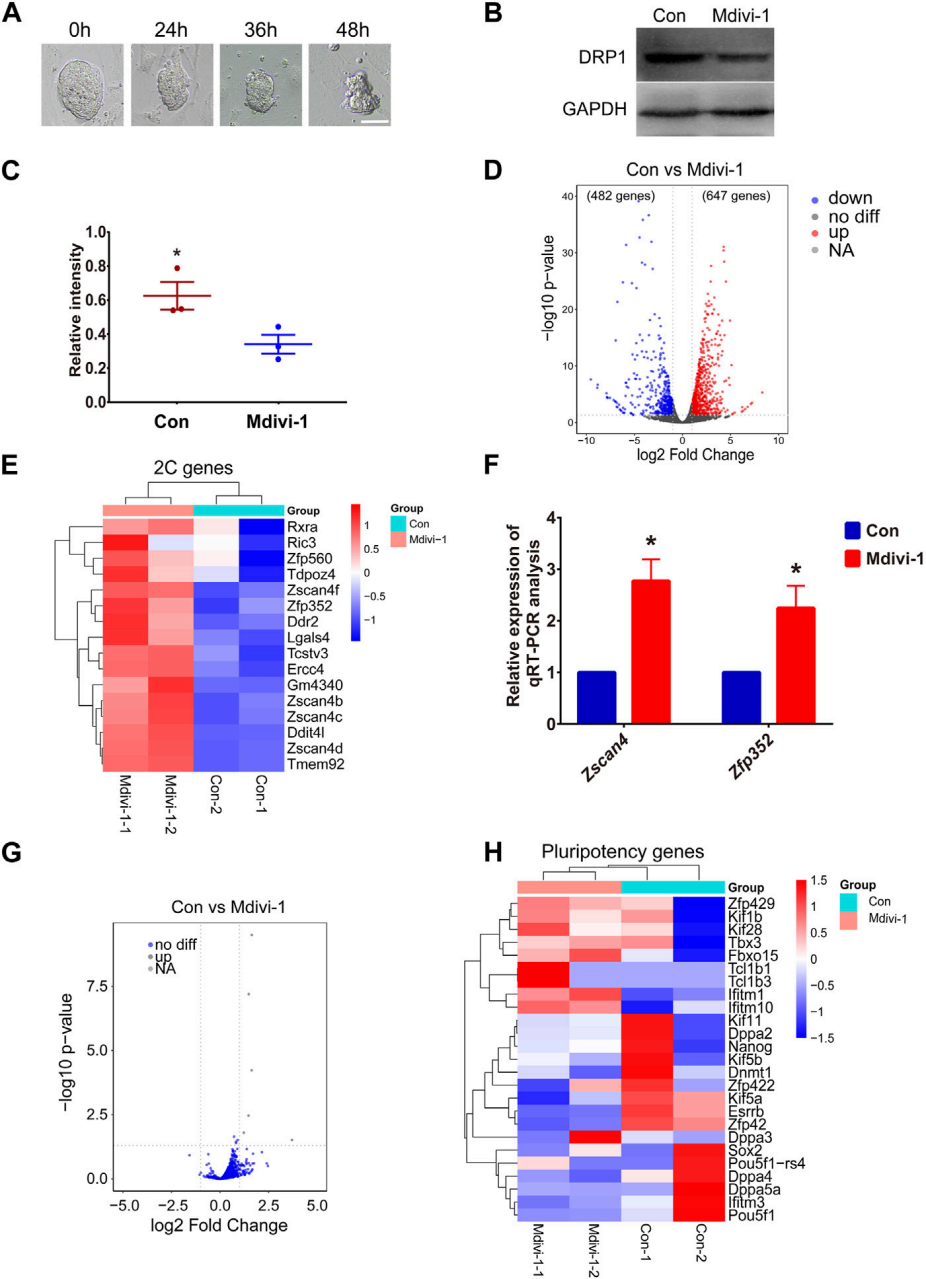
The expression level of 2C genes in Mdivi-1-treated ESCs increased compared to control ESCs. **(A)** DIC images of ESCs in 50 µM Mdivi-1 treatment after culturing for 0, 24, 36, and 48 h, respectively. Scale bar, 50 µm. **(B, C)** Western blotting of DRP1 and the statistical analysis of DRP1 protein level in control and Mdivi-1-treated ESCs. GAPDH was used as the internal control. *p < 0.05. **(D)** Volcano plot comparing differentially expressed genes (1129 genes) between Mdivi-1-treated ESCs and the control group. The value of |log2 fold-change| to mark the upregulated (red) (647 genes) and downregulated (blue) (482 genes) genes. **(E)** Heatmap of differential expression of 2C genes in Mdivi-1-treated mESCs compared to the control group. **(F)** The relative expression of *Zscan4*, *Zfp352,* mRNA in control and Mdivi-1-treated ESCs. *p < 0.05. **(G)** Volcano plot shows differential expression of transposons in control and Mdivi-1 treatment ESCs. **(H)** Heatmap of differentially expressed genes of pluripotent genes in control and Mdivi-1-treated ESCs.

Mdivi-1 is a cellular permeable selective inhibitor of DRP1. In this study, we inhibited the activity of DRP1 in mouse ESCs by adding Mdivi-1 to the ESC medium. We first treated ESCs with Mdivi-1 at different concentrations for 24 h. However, when the drug concentration was very high, the morphology of ESC clones worsened and the cells died (Supplementary Figure S1). After comparison, we treated mouse ESCs with a concentration of 50 μM. We then examined the effects of drug treatment time on stem cell status. However, ESCs gradually died after treatment with 50 μM Mdivi-1 for more than 24 h (Figure 2A). Therefore, in this study, we treated ESCs with 50 μM Mdivi-1 for 24 h for subsequent detection. Western blot analysis confirmed that the expression of DRP1 was decreased after treatment of ESCs with Mdivi-1 (Figures 2B, C). Next, RNA-seq data on ESCs before (control group) and after (treatment group) Mdivi-1 treatment were analyzed. Compared with the control group, there were 1,129 differentially expressed genes in the treatment group, among which 647 genes were upregulated and 482 genes were downregulated (Figure 2D). Among these significantly upregulated genes, we noticed several typical 2C genes (Figure 2E and Supplementary Figure S2A). Moreover, the results of qRT-PCR proved that 2C genes such as *Zscan4* and *Zfp352* were indeed significantly upregulated (Figure 2F). In addition, we noticed no significant changes of transposon expression upon Mdivi-1 treatment (Figure 2G). Notably, our results also showed no significant changes in the expression of pluripotent genes in Mdivi-1-treated ESCs (Figure 2H). These results implied that the transition of totipotency-to-pluripotency may be disrupted by the abnormal activity of mitochondria, resulting in failed silencing of 2C genes.

**FIGURE 3:**
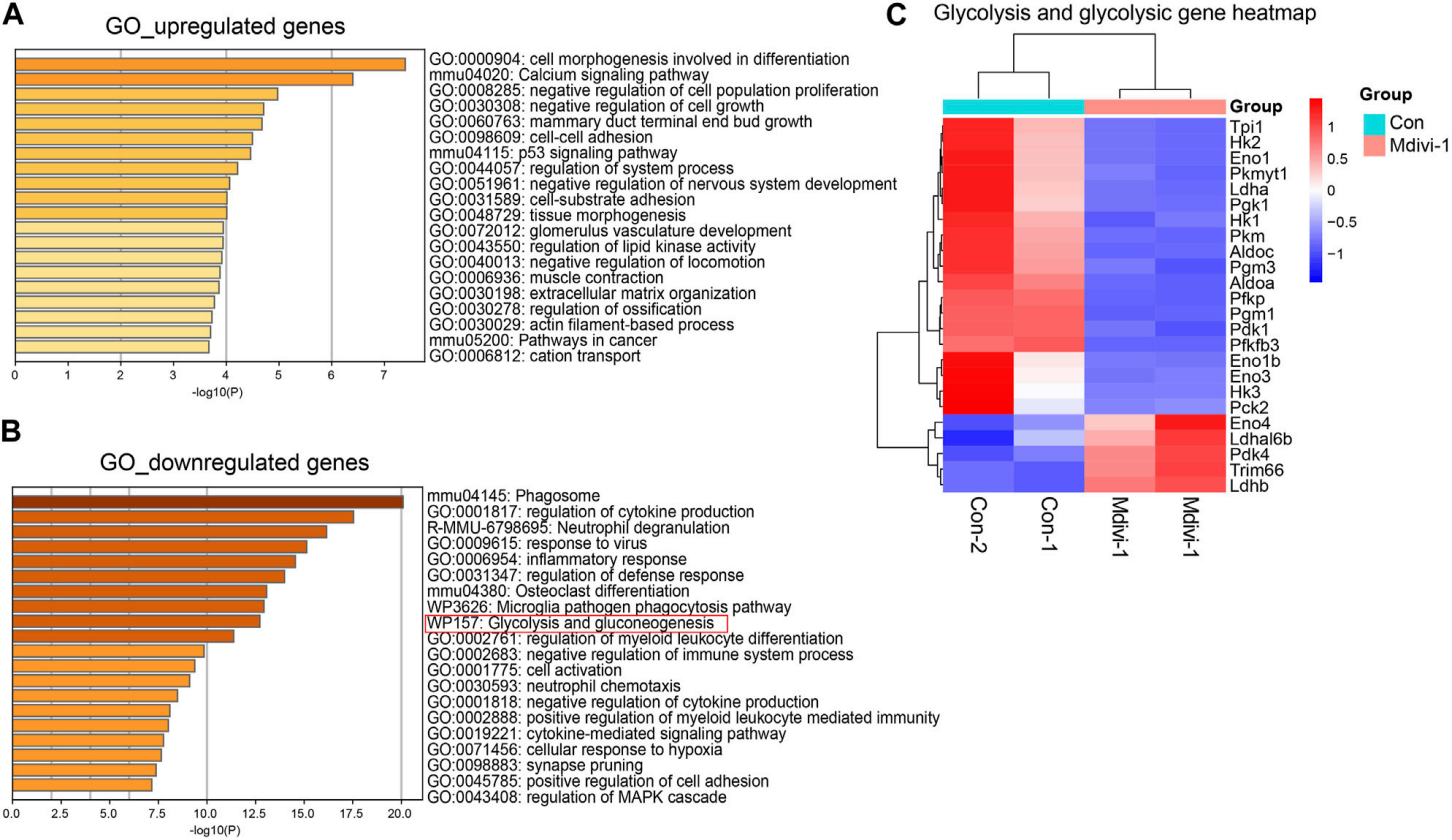
Schematic diagram of RNA-seq data analysis. GO enrichment analysis of upregulated **(A)** and downregulated genes **(B)** in Mdivi-1-treated ESCs compared to the control group. **(C)** Heatmap of differentially expressed genes of glycolysis/gluconeogenesis genes in control and Mdivi-1-treated ESCs.

In addition, gene enrichment analysis showed that these upregulated genes were enriched in “p53” and other pathways (Figure 3A). Studies have shown that P53 promoted *Dux*-mediated 2C gene upregulation (Li et al., 2021). Subsequently, we found that the downregulated genes were enriched in the “glycolysis/gluconeogenesis” pathway (Figure 3B). In these differentially expressed genes, multiple gene encoding enzymes in the glycolysis/gluconeogenesis pathway were downregulated (Figure 3C). These results showed that the glycolysis activity of Mdivi-1-treated cells might be decreased. Previous reports showed that glycolysis activity in *Drp1*-KO ESCs was indeed decreased (Seo et al., 2020), supporting our discovery. Decreased glycolysis pathway activity promoted the activation of the 2C program in ESCs (Hu et al., 2020). In conclusion, inhibition of DRP1 by Mdivi-1 can promote the expression of 2C genes by changing a variety of biological pathways in mouse ESCs.

### Inhibition of DRP1 facilitated the transcriptional activity of 2C genes

**FIGURE 4:**
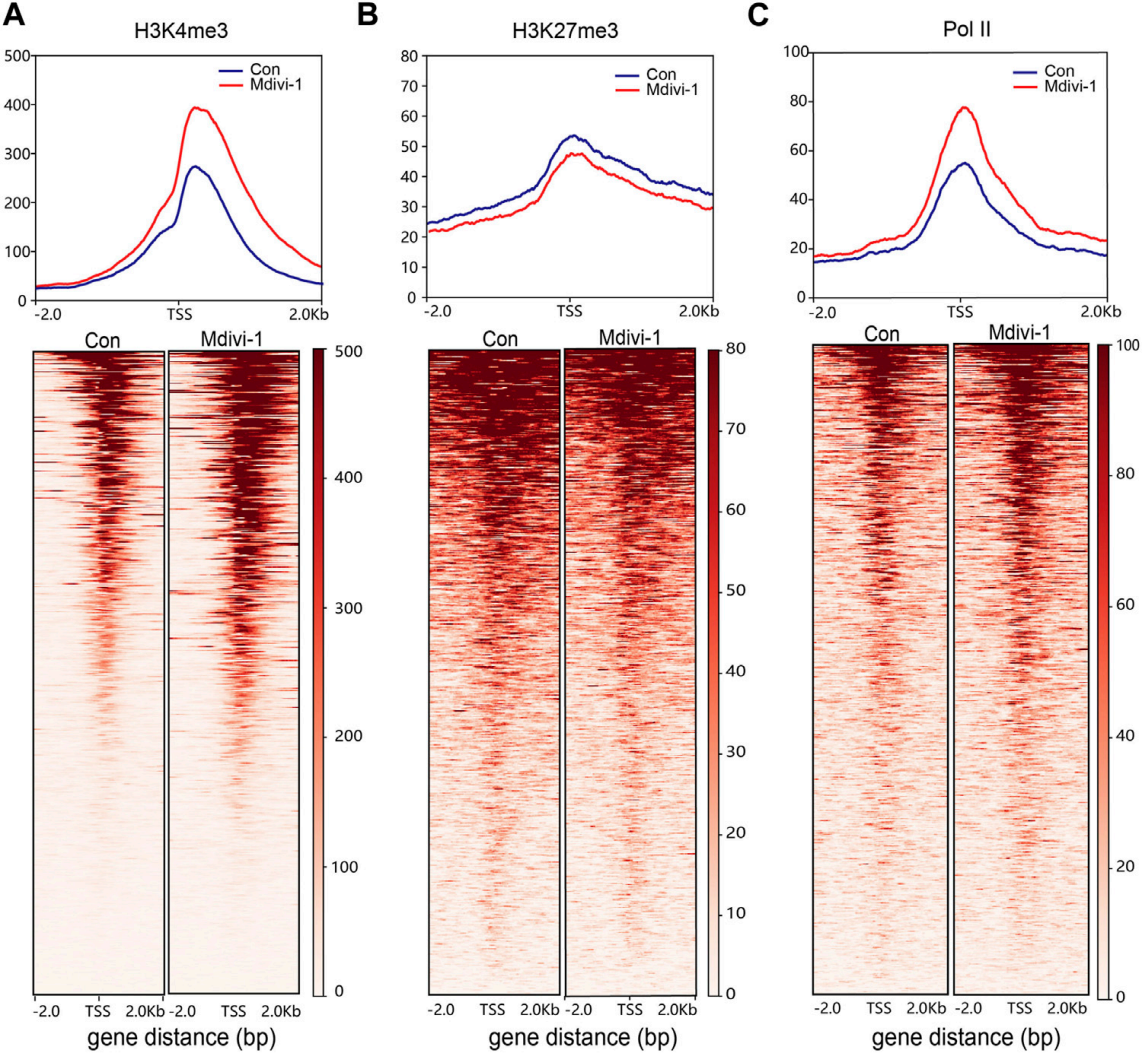
Comparison of H3K4me3, H3K27me3, and Pol II occupancy. The signal intensity diagram of H3K4me3 **(A)**, H3K27me3, **(B)** and Pol II **(C)** at gene promoters (TSS flanking 2 kb) of upregulated genes in the Mdivi-1-treated group compared to the control group (upper) and the heatmap of upregulated genes at gene promoter regions (lower).

To further understand the mechanism of 2C gene upregulation, we detected the enrichment of H3K4me3, H3K27me3, and RNA polymerase II (Pol II) in mouse ESCs after Mdivi-1 treatment. We found that H3K4me3, a marker of transcription initiation, had significantly increased accumulation at transcription start sites (TSSs) of upregulation genes (Figure 4A). In contrast, H3K27me3 was considered a marker of transcriptional inhibition, and accumulation at TSSs of upregulated genes was significantly reduced (Figure 4B). At the same time, we found that Pol II occupation around the upregulated TSSs was increased after Mdivi-1 treatment (Figure 4C). These results suggest that inhibition of DRP1 expression by Mdivi-1 can enhance transcriptional activity by strengthening H3K4me3 and reducing H3K27me3 at TSSs, thereby promoting 2C gene expression.

### Inhibition of DRP1 led to failed silencing of 2C genes in late 2C embryos

**FIGURE 5:**
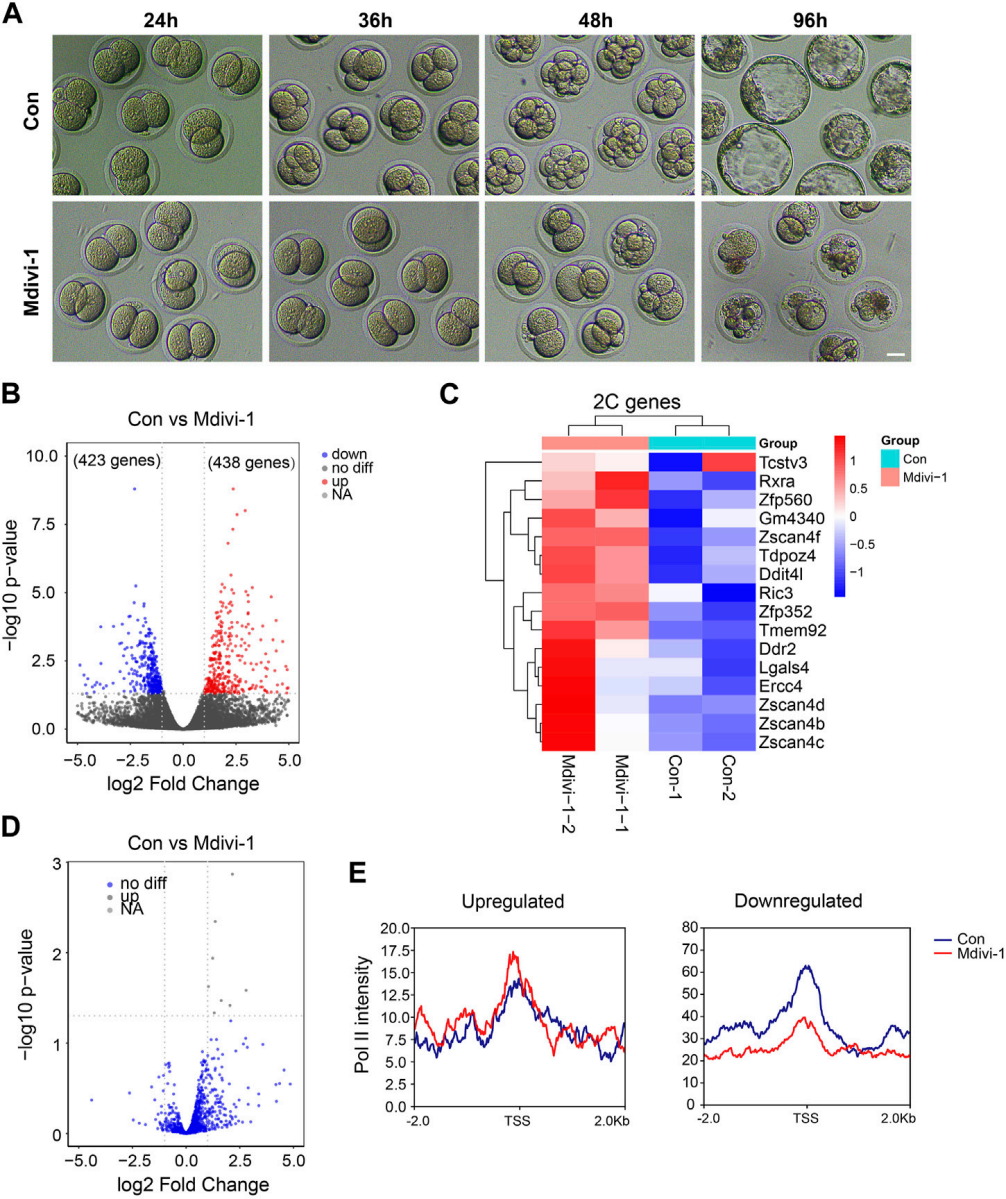
Mdivi-1 treatment impacted 2C gene expression in late 2-cell mouse embryos. **(A)** DIC images of early embryonic development in control and 200 µM Mdivi-1-treated groups after culturing for 24, 36, 48, and 96 h, respectively. Bar = 20 µm. **(B)** Volcano plot showing differentially expressed genes (861 genes) between Mdivi-1-treated embryos and control embryos. |log2 fold-change| marks the upregulated (red) (438 genes) and downregulated (blue) (423 genes) genes. **(C)** Heatmap of differential expression of ZGA genes in Mdivi-1-treated embryos compared to control embryos. **(D)** Volcano plot showing the expression changes of transposons in Mdivi-1-treated mouse embryos compared with control embryos. **(E)** Signal intensity diagram of Pol II at TSSs of upregulated and downregulated genes in Mdivi-1-treated group (red) compared to the control group (blue).

To identify how DRP1 activity is involved in the cell fate control in early mouse embryos, we treated mouse early embryos with Mdivi-1 at the late zygote stage when embryonic transcription started. After culturing the embryos for 96 h, we noticed significant developmental delay upon Mdivi-1 treatment (Figure 5A). The RNA-seq analysis of late 2-cell (2C) embryos showed that 861 genes were differentially expressed in the Mdivi-1-treated group when compared with the control group (Figure 5B). Among the 861 DEGs, 438 genes were overexpressed and 423 genes were downregulated in late 2C embryos. Interestingly, we found the 2C genes, such as *Zfp352* and *Zscan4d*, were upregulated in Mdivi-1-treated embryos (Figure 5C and Supplementary Figure S2B), and this was similar to what happened in mouse ESCs. We also found no general differences in retrotransposon expression at the late 2C stage after DRP1 inhibition, which was also similar to that in mouse ESCs (Figure 2G; Figure 5D). In conclusion, inhibition of DRP1 can effectively promote the expression of 2C genes in late 2C embryos, while 2C genes are supposed to be silenced at this stage to start mid-implantation development and facilitate totipotency-to-pluripotency transition. In addition, the enrichment of Pol II at the differentially expressed genes in the late 2C stage with and without Mdivi-1 treatment was also examined. As expected, Pol II had significantly increased accumulation at TSSs of upregulated genes in late 2C embryos upon Mdivi-1 treatment (Figure 5E). In contrast, the enrichment of Pol II at TSSs of these downregulated genes was reduced obviously (Figure 5E).

Taken together, our results showed that DRP1 activity is important in early mouse embryos to silence 2C genes at the late 2C stage to drive developmental programs for the totipotency-to-pluripotency transition.

## Discussion

A transient ESC subpopulation displays totipotent features by expressing a set of genes, which are activated in 2C embryos (Le et al., 2020). In the mouse ESC culture medium, the 2C-like state is transient and can be transformed back into pluripotency, and the process is dynamic and reversible (Fu et al., 2020). Studies have shown that pluripotent stem cells and 2C-like cells have different metabolic pathways (Zhao et al., 2021). Mitochondrial function maintained the proliferation of self-renewing ESCs (Mandal et al., 2011). RNA-seq analysis shows knocked-out mitochondrial fission-related factors, such as *Drp1*, *Fis*, or *Mff*, does not affect the expression of pluripotent genes in ESCs (Seo et al., 2020). However, we noticed that the 2C gene levels were increased in KO-ESCs (Seo et al., 2020). In our study, the mitochondrial fission inhibitor Mdivi-1 was used to effectively inhibit DRP1 activity, even at the protein level. In addition, treatment with Mdivi-1 could effectively increase the expression of 2C genes such as *Zscan4* and *Zfp352* in ESCs. This is in agreement with the result from *Drp1*-KO ESCs. It was suggested that the cellular pluripotency transformation disorder appeared due to inhibition of mitochondrial activity, which resulted in failed silencing of totipotency-related genes.

In this study, we identified that Mdivi-1 treatment facilitated the expression of 2C genes through accumulated H3K4me3 modification and reduced H3K27me3 modification. H3K4me3 is associated with gene activation (Liu et al., 2016). It is reported that highly upregulated 2C genes are correlated with the deposition of H3K4me3 (Zhang et al., 2021). The proportion of H3K4me3 tended toward TSSs was significantly increased in 2C-like cells compared with ESCs, which is similar to 2C embryos (Zhang et al., 2021). In our previous study, we noticed disturbed ZGA events in early 2C embryos treated with Mdivi-1 (Li et al., 2021). Interestingly, 2C genes were increased in late 2C embryos upon Mdivi-1 treatment. The role of retrotransposons in the regulation of transcription cannot be ignored, but our analysis results showed that the changes in transposons were not obvious, indicating that the upregulation of 2C genes may not be regulated by transposons. We propose that it was due to important roles of mitochondrial fission not only in totipotency establishment but also in the totipotency-to-pluripotency transition.

GO analysis in this study showed that the activity of the glycolysis/gluconeogenesis pathway decreased upon DRP1 inhibition, and the glycolysis/gluconeogenesis pathway-related enzymes were indeed significantly downregulated. Many stem cells including ESCs appear to be more dependent on glycolysis to produce adenosine-5 ′-triphosphate (ATP) than differentiated cells, and this phenomenon is similar to cancer cells (Rafalski et al., 2012). The highly active glycolysis pathway maintains the expression of pluripotent factors and the pluripotent state of mouse ESCs (Mandal et al., 2011). Notably, glycolysis participation is a key step in the transformation of terminally differentiated cells into induced pluripotent stem cells (iPSCs) (Varum et al., 2009; Rafalski et al., 2012). The expression level of pluripotency markers was increased when the activity of mitochondria was inhibited by chemical conditions, while pluripotency gene expression was not significantly affected in ESCs after Mdivi-1 treatment in our study. In *Drp1*-KO ESCs, the decreased activity of the glycolysis pathway was found, but there was no significant difference in the expression of pluripotent factors (Seo et al., 2020). Related studies have shown that the inhibition of the glycolysis pathway can promote the expression of 2C genes in ESCs (Hu et al., 2020). Pyruvate produced by the glycolysis pathway will participate in the mitochondrial TCA cycle. Acetyl-CoA is produced by mouse ESCs through the action of threonine dehydrogenase as an important source of energy metabolism (Mandal et al., 2011; Shyh-Chang et al., 2013). In addition to the downregulation of the glycolysis/gluconeogenesis pathway, a decrease in lipid kinase may lead to a decrease in lipid metabolic efficiency. Recent studies indicated that lipid metabolism has gradually become a critical regulator of stem cell differentiation (Mandal et al., 2011; Knobloch et al., 2013). The products of lipid metabolism also eventually participate in the TCA cycle. Although we did not find the significant changes in the TCA cycle in GO analysis, RNA-seq analysis showed that the expression of *Pck2* was downregulated. Therefore, we hypothesized that decreased glycolytic pathway activity caused by abnormal mitochondrial fission would indirectly promote the upregulation of 2C genes in ESCs. This may be due to the change in metabolite quantity in the glycolysis/gluconeogenesis pathway, which indirectly affects TCA products and 2C gene expression. This deserves further experimental verification.

## Conclusion

Our results showed that inhibition of DRP1 induced the increased levels of 2C genes in both mouse ESCs and mouse late 2C embryos, which may be due to the disturbed transition from totipotency-to-pluripotency status. After Mdivi-1 treatment, the intensity of H3K27me3, which is associated with gene repression, was decreased at the TSSs of upregulated genes in ESCs. In contrast, increased H3K4me3 occupancy and Pol II deposition at TSSs were observed. Therefore, inhibition of DRP1 stimulated H3K4me3 and Pol II accumulation at 2C genes. In addition, these results implied the change in metabolite quantity in the glycolysis/gluconeogenesis pathway may indirectly affect TCA products and the expression levels of 2C genes.

## Data Availability

The datasets presented in this study can be found in online repositories. The names of the repository/repositories and accession number(s) can be found at: https://www.ncbi.nlm.nih.gov/geo/query/acc.cgi?acc=GSE221989.
