# Supplementary material for: Regulation of cleavage embryo genes upon DRP1 inhibition in mouse embryonic stem cells
Source: Front Cell Dev Biol. 2023 May 15;11:1191797. doi: 10.3389/fcell.2023.1191797 (PMC10225531; doi:10.3389/fcell.2023.1191797)
Supplement: Supplementary file 2 [file DataSheet1.docx]

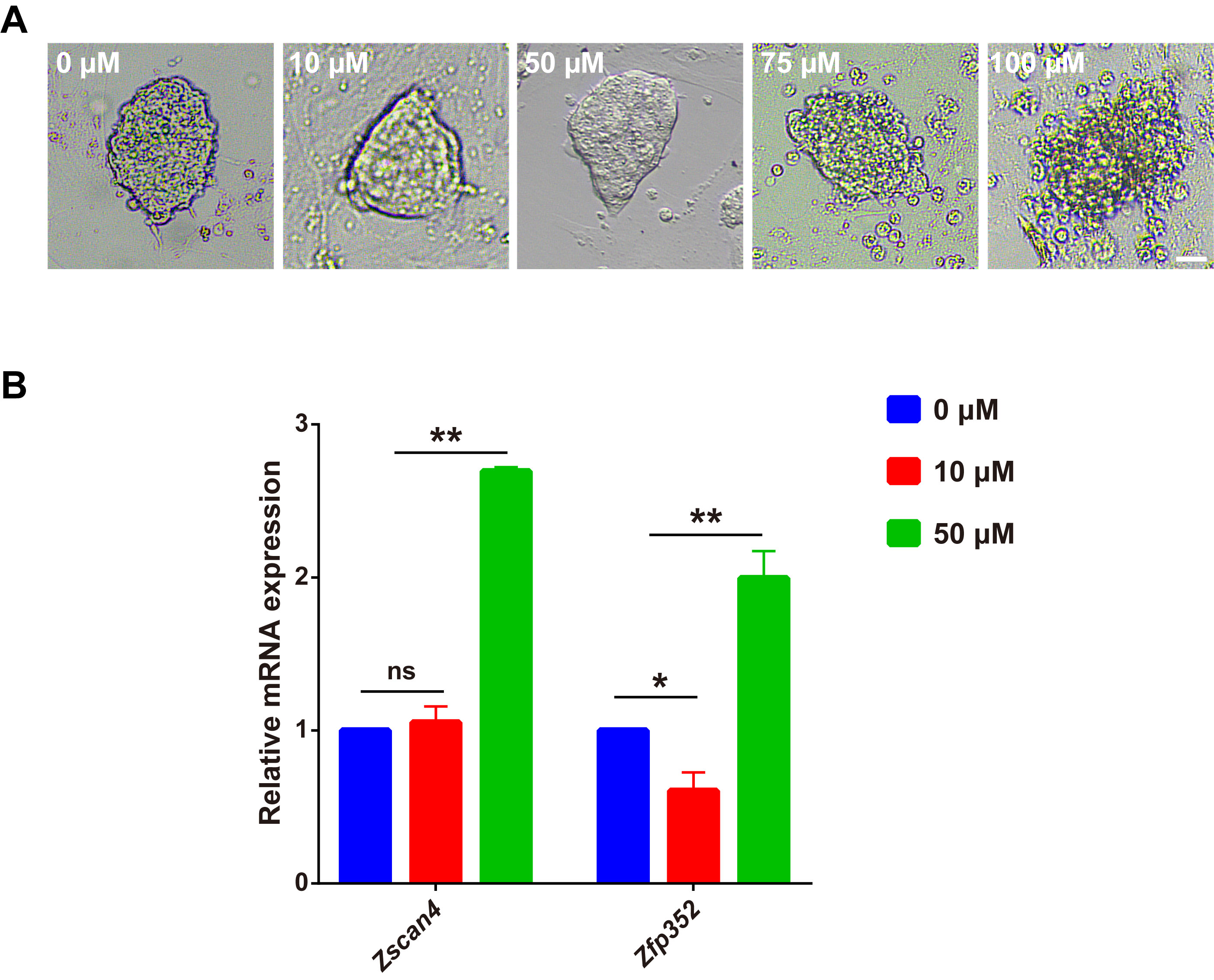


**Fig. S1. Effect of different concentration of Mdivi-1 on ESCs.** (A) DIC images of ESC in 0, 10, 50, 75 and 100 µM Mdivi-1 treatment after culturing for 24 h, respectively. Scale bar, 50 µm. (B) The relative expression of *Zscan4* and *Zfp352* mRNA in 0, 10, and 50 µM Mdivi-1 treatment ESCs. * P < 0.05, ** P < 0.01.


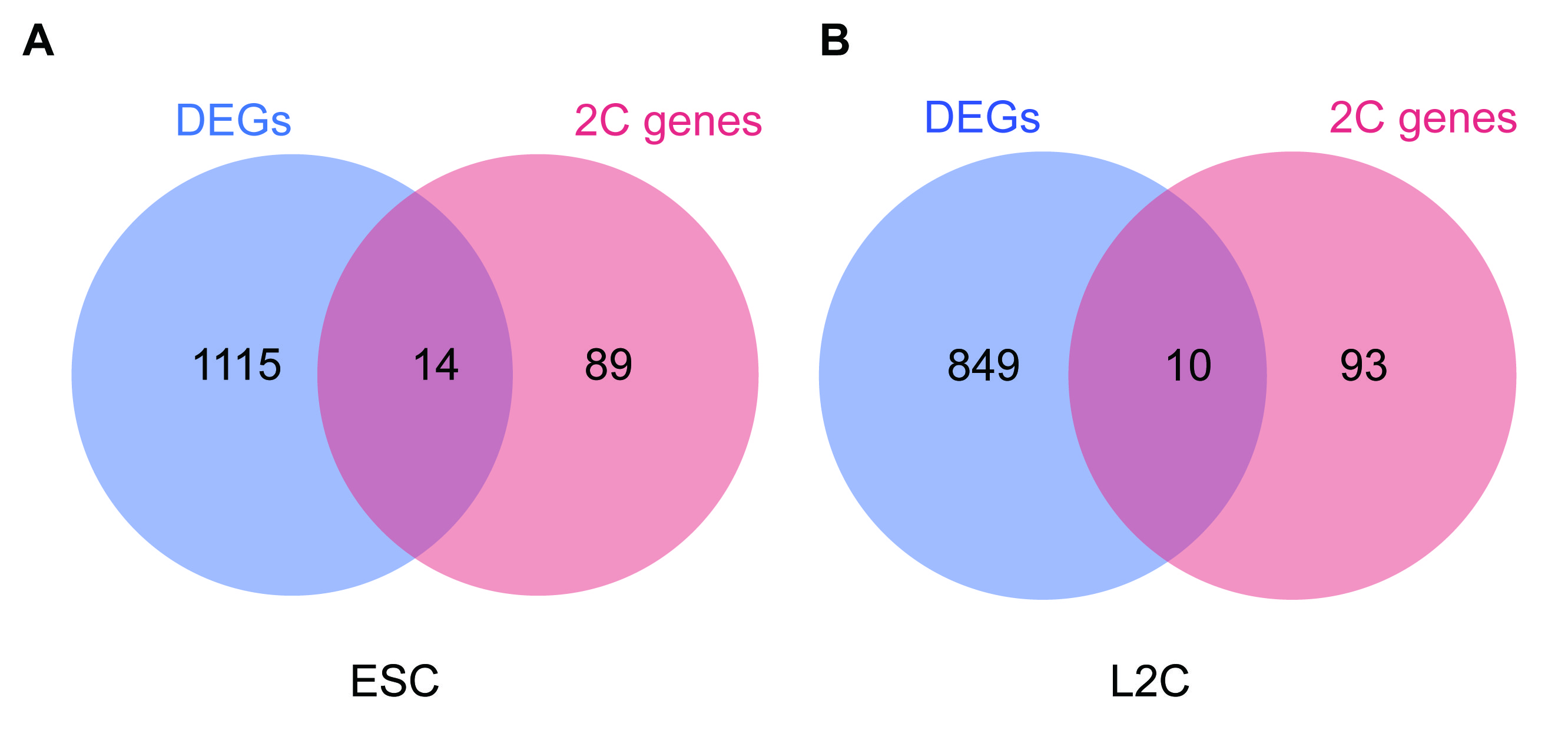


**Fig. S2.** Venn diagram showing the overlap of the differential expressed genes and all 2C genes in Mdivi-1 treated ESCs (A) and Mdivi-1 treated mouse embryos (B).
